# Pharmacological CCR1 blockade limits infarct size and preserves cardiac function in a chronic model of myocardial ischemia/reperfusion

**DOI:** 10.1186/cc9424

**Published:** 2011-03-11

**Authors:** A Van de Sandt, S Zander, S Becher, R Ercan, C Quast, J Ohlig, T Lauer, T Rassaf, M Kelm, MW Merx

**Affiliations:** 1 Department of Cardiology, Pulmonary Diseases and Vascular Medicine, University Hospital, Düsseldorf, Germany

## Introduction

This study sought to determine the chronic effects of pharmacological blockade of the chemokine receptor CCR1 via application of the potent, selective antagonist BX471 in a murine model of myocardial ischemia/reperfusion (I/R). CCR1 is a prominent receptor in mediating inflammatory leukocyte recruitment. The intense inflammatory response is considered to be a key component of cardiac remodelling. Thus, limiting the post-reperfusion inflammatory pattern seems to be a promising therapeutic approach in limiting reperfusion injury. Previously, we demonstrated that CCR1^-/- ^mice exhibit attenuated infarct expansion and preserved LV function in a chronic model of myocardial no-reflow infarction due to an abrogated inflammatory response.

## Methods

C57/B6 mice underwent a 60-minute coronary occlusion in a closed-chest model of myocardial I/R. Mice were treated with the specific CCR1 antagonist, BX471 (50 mg/kg BW, s.c.), or placebo, for 96 hours at 8-hour intervals starting 15 minutes prior to reperfusion. At 21 days of reperfusion, cardiac function was assessed using a pressure- volume catheter (Millar) inserted in the left ventricle. Infarct size was analysed and cardiac content for collagen was elucidated.

## Results

Infarct size was significantly smaller in the BX471-treated group (placebo: 20.7 ± 2.8% vs. BX471: 11.6 ± 4.2%, *P *< 0.05; area at risk did no differ between the groups). At 21 days of reperfusion BX471-treated mice exhibited a tendency towards improved cardiac function. Significantly improved diastolic function was documented in BX471-treated mice (d*P*/d*t*_min _placebo: -7,635 ± 1,090 vs. BX471: -9,845 ± 657, *P *< 0.01). In histochemical analysis, collagen content was elevated in the hearts of BX471-treated mice.

## Conclusions

Pharmacological CCR1 antagonism leads to improved diastolic function and attenuated infarct size in a chronic model of ischemia/reperfusion, suggesting that CCR1 antagonism might provide a promising therapeutic approach in myocardial infarction. The increased cardiac collagen documented in the treated group of our study might point towards a beneficial effect in the restructuring of the extracellular collagen matrix. Further studies of the underlying mechanisms and a detailed analysis of structural remodelling after pharmacological CCR1 blockade are warranted.

